# Temporal Pattern of Individual Neurological Function Recovery in Guillain–Barré Syndrome

**DOI:** 10.3390/jcm13185635

**Published:** 2024-09-23

**Authors:** Roopali Mahajan, Jayantee Kalita, Vishal Jha, Nagendra B. Gutti, Prakash C. Pandey, Usha K. Misra

**Affiliations:** Department of Neurology, Sanjay Gandhi Post Graduate Institute of Medical Sciences, Raebareli Road, Lucknow 226014, India; mahajanroopali007@gmail.com (R.M.); vishaljha86@gmail.com (V.J.); drnag1289@gmail.com (N.B.G.); drprakashpandey@gmail.com (P.C.P.); drukmisra@rediffmail.com (U.K.M.)

**Keywords:** Guillain–Barré syndrome, axonal, demyelinating, AMAN, AIDP

## Abstract

**Background**: There is paucity of studies on the temporal pattern of recovery of facial, bulbar, sensory, motor, and autonomic dysfunction in Guillain–Barré syndrome (GBS), although many studies have reported short- and long-term functional outcomes. We report the temporal pattern of recovery of various neurological functions in GBS, and compare the pattern of recovery between acute inflammatory demyelinating polyradiculoneuropathy (AIDP) and acute motor axonal neuropathy (AMAN). **Methods**: Forty-two patients with GBS were prospectively included, and their clinical details, including peak disability on a 0–6 scale, were noted. The day of complete recovery in motor, sensory, facial, bulbar, and autonomic functions during 3 months of follow-up was recorded. **Results**: Complete recovery of autonomic function occurred in all (median, 12 days), bulbar weakness in 91.3% (median, 15 days), facial weakness in 86.2% (median, 19 days), and sensory functions in 82.1% (median, 20 days). Only 9.5% of patients achieved normal motor function within 3 months. The days of complete recovery of bulbar, facial, autonomic, and motor deficits were comparable between AIDP and AMAN. Demyelinating GBS had an earlier recovery of bulbar and sensory functions. **Conclusions**: The neurological recovery in GBS occurs first in the autonomic, followed by the bulbar, facial, sensory, and motor functions. The demyelinating type had an earlier recovery of bulbar and sensory functions.

## 1. Introduction

Guillain–Barré syndrome (GBS) is a rapidly progressive flaccid quadriparesis that occurs within 4 weeks with or without sensory deficit, cranial nerve palsy, or autonomic dysfunction, and it is associated with albumino-cytological dissociation in cerebrospinal fluid (CSF). The incidence of GBS is 0.81–1.91 cases/100,000 person years in Europe and North America [1,2,3]. Based on nerve conduction studies, four distinct subtypes of GBS have been described: acute inflammatory demyelinating polyradiculoneuropathy (AIDP), acute motor axonal neuropathy (AMAN), acute motor sensory axonal neuropathy (AMSAN), and inexcitable motor nerve (IEMN) [4]. During the course of the illness, however, the electrodiagnostic (EDx) subtypes shift from one to another [5]. For classification and prognosis, the EDx at one month has been more useful [6,7]. The temporal profile of recovery of different neurological functions, such as the motor, sensory, cranial nerve, and autonomic, may have a different trajectory. Axonal regeneration occurs at a rate of 1 mm/day; thereby, it is likely for recovery to take a longer time in an axonal form of GBS such as AMAN, AMSAN, and IEMN. Rapid recovery, however, has been reported in 11–40.9% patients with AMAN [8,9]. Some authors have reported consistent recovery in AIDP, and others have reported a recovery pattern similar to AMAN [10,11]. There is a paucity of studies evaluating the temporal pattern of recovery of various neurological functions in GBS, although there are studies on long-term prognostic predictors [12,13]. We hypothesise that shorter cranial nerves may improve early, and the demyelinating (AIDP) may have an earlier recovery of motor and autonomic functions than the axonal (AMAN) variety. In this study, we report the pattern of motor, sensory, autonomic, and cranial nerve recovery in the patients with GBS, and compare the recovery pattern between AMAN and AIDP, as well as the axonal and demyelinating subtypes.

## 2. Materials and Methods

Consecutive patients with GBS admitted to our service during 2022 to 2023 were prospectively included. The diagnosis of GBS was based on clinical data, nerve conduction study, and CSF findings [14]. The patients with coexistent neuropathy due to other illnesses such diabetes mellitus, HIV, chronic renal or hepatic failure, vasculitis, toxins, drugs, and porphyria were excluded.

The study protocol was approved by the Institute Ethics Committee (IEC code: PGI/BE/296/2022 SGPGIMS, Lucknow, India), and the patients consented to the study.

### 2.1. Clinical Evaluation

A detailed history was ascertained, including demographic information, duration of symptoms, day of hospitalisation from the symptom onset, and peak disability. The clinical disability was graded on a 0–6 GBS disability scale (GBSDS) as follows [15]:

0 = Healthy

1 = Minor symptoms and signs, and could do manual work;

2 = Could walk unaided but unable to do manual work;

3 = Could walk with a stick, appliance, or support;

4 = Confined to bed or bedbound;

5 = Requires assisted ventilation;

6 = Dead.

The triggering events (diarrhoea, fever, respiratory tract infection, vaccination etc.) in the previous 6 weeks were noted. Symptoms related to cranial nerve palsy including visual impairment, diplopia, ptosis, ophthalmoplegia, facial asymmetry, inability to close eyes, facial numbness, chewing abnormality, nasal intonation of voice, nasal regurgitation, choking, and tongue weakness were enquired about. Symptoms related to autonomic dysfunction (palpitation, excessive sweating, hypertension, dryness of mouth) were also noted. Bedside autonomic function tests were evaluated [resting tachycardia; loss of sinus arrhythmia, and postural hypotension (>20 mmHg of fall on upright position)]. A cardiac monitor was used for assessing variabilities in heart rate and blood pressure [16]. Muscle power was assessed using the Medical Research Council Power Scale [17]. The abnormality in muscle tone and tendon reflex was categorised as normal, hypo, or hyper. Sense of pin prick was assessed using a sterilised pin and fine touch by a feather. Joint position and movements were assessed using 1° deflections of great toe and thumb. If impaired, proximal joints were also tested. Vibration was tested by putting a 128 Hz tuning fork over the bony prominences, from distal to proximal sites.

### 2.2. Laboratory Investigations

Blood counts, haemoglobin, erythrocyte sedimentation rate at one hour, blood glucose, serum creatinine, transaminases, bilirubin, potassium, sodium, albumin, calcium, and alkaline phosphatase were estimated. A radiograph of the chest and electrocardiogram were performed. HIV serology and urine porphobilinogen were conducted. Lumbar cerebrospinal fluid analysis was performed for cells, protein, and glucose.

*Nerve conduction studies*: Nerve conduction studies (NCS) were performed at admission, and repeated after 3–4 weeks, if the initial NCS was normal or equivocal. Motor nerve conduction studies of the median, ulnar, and common peroneal nerves were conducted using the standard technique. The distal motor latency, distal compound muscle action potential (CMAP), conduction velocity, conduction block, and minimal F wave latencies were noted. Sensory nerve conduction studies of the median, ulnar and sural nerves were performed on both sides using standard techniques. The sensory nerve conduction velocity and base to peak amplitude were measured. The neurophysiological findings were compared with our laboratory normative data while reporting the test [18].

### 2.3. Subtypes of GBS

Neurophysiological subtypes were analysed considering the NCS findings into AIDP, AMAN, AMSAN, IEMN, equivocal, and normal. GBS patients were categorised as AIDP if the NCS revealed at least one demyelinating feature in at least two nerves (prolonged distal motor latency, conduction block, the slowing of conduction velocity, or prolonged minimal F latency) or ≥2 demyelinating features in one nerve, if all other motor nerves were inexcitable or the CMAP (compound muscle action potential) was ≥10% of the lower limit of normal [4]. The NCS findings for the diagnosis of the AMAN subtype included a reduced CMAP (<80% of LLN) or unrecordable motor NCS in ≥2 nerves with normal sensory conduction. More than one demyelinating feature in the NCS was not allowed in AMAN. The diagnostic criteria of AMSAN were like AMAN, with a reduced SNAP (sensory nerve action potential) in ≥2 sensory nerves, with a marginal slowing in conduction velocity [19]. The NCS criteria of IEMN were an absent distal CMAP in all motor nerves. Only one motor nerve could have a recordable distal CMAP of <10% of LLN in IEMN [4]. Nerve conduction studies not fitting into the above subtypes were considered equivocal.

### 2.4. Treatment

The patients with a GBSDS of more than 2 received IVIg (400 mg/kg/day × 5 days) within 15 days of illness, or received plasmapheresis (PLEX, 30 mL/kg/day × 5 days) within 1 month of illness. Supportive care was given to all. The patients with respiratory failure were intubated and mechanically ventilated. Those with bulbar weakness were fed by nasogastric tube. A beta-blocker (propranolol) 10–20 mg was prescribed to the patients with sympathetic overactivity, and these patients were on cardiac monitoring.

### 2.5. Follow-Up

Patients were discharged from the hospital once the disease condition was stabilised, especially respiratory and autonomic functions. They were physically followed up at one month and three months. Patients and their first-degree relatives were advised to note the day of normalisation of facial (eye closure and lip closure), bulbar (normal swallowing of liquid and solid), motor (ability to get up from sitting, climbing up and down stairs, walking on heels and toes, breaking chapati and combing hair) and sensation (touch, temperature, and pin prick) function. During the hospital stay, patients’ attendants were trained to conduct these tests. In patients with dysautonomia, we recorded the maximum and minimum heart rate and blood pressure, an electrocardiogram for respiratory variation, and postural drop of blood pressure during the hospital stay. The attendant of the patients was taught to measure the blood pressure and pulse using a digital blood pressure instrument, and upload the document to a dedicated GBS WhatsApp group, which was monitored by the authors (JK, RM and VJ). Patients were considered to have an early recovery if the GBSDS improved by a >2 grade within two weeks of the nadir. The outcome at three months was defined as good (GBSDS ≤ 2), poor (GBSDS 3–5), or death.

### 2.6. Statistical Analysis

The categorical data are expressed as frequency and continuous data as mean ± standard deviation. The baseline characteristics and pattern of recovery of motor, facial, bulbar, and autonomic functions were compared between AIDP and AMAN using a chi square (categorical) or independent *t* test (normally distributed continuous variable)/Wilcoxin signed-rank test (not normally distributed continuous variable). A similar comparison was also performed for patients with the demyelinating (AIDP, equivocal and normal) and axonal (AMAN, IEMN, and AMSAN) subtypes. A Kaplan–Meier analysis was conducted for the recovery of facial, bulbar, autonomic, sensory, and motor functions between the patients with the demyelinating and axonal subtypes, as well as between the AIDP and AMAN subtypes. A two-tailed *p* value of ≤0.05 was considered significant. SPSS 20 software was used for the statistical analysis, and graphs were prepared using GraphPad Prism 5 and R software (R Core Team (2022)).

## 3. Results

Forty-six patients with GBS were admitted during the study period, and four were excluded because of systemic lupus erythematosus (*n* = 1), leukaemia (*n* = 2), and lymphoma (*n* = 1). The results therefore are based on 42 patients. Their median age was 31 years, and 11 (26.2%) were females. Thirty patients (71.4%) were admitted within seven days of illness. Twenty-five (59.5%) had dysautonomia, 29 (69%) had facial palsy, 23 (54.7%) had bulbar palsy, and 7 required mechanical ventilation. The commonest EDx subtype was AIDP (25, 59.5%), followed by AMAN (10, 23.8%), IEMN (4, 9.5%), and AMSAN, equivocal and normal in 1 patient (2.4%) each. The detailed clinical information is summarised in Supplementary Table S1.

### 3.1. Pattern of Recovery

**Figure 1:**
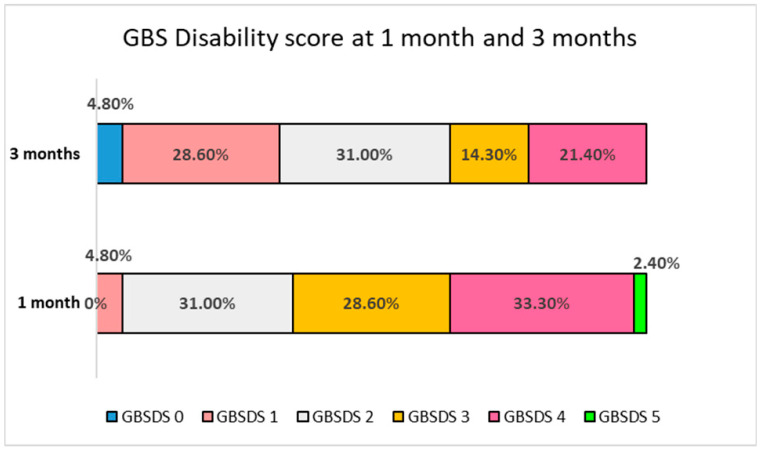
Bar diagram shows proportion of patients having GBSD score at one and three months.

During the three months follow-up, all the patients had some improvement. All 42 patients had a complete recovery of autonomic functions. A total of 91.3% had a recovery of bulbar function, and 86.2% recovered in facial palsy, 82.1% patients regained normal sensation in joint position and pin prick sensation. The complete recovery of motor function, however, occurred in two (4.7%) patients only. At 3 months, the GBSDS was zero in 2 (4.7%), 1 in 12 (28.5%), 2 in 13 (30.9%) and ≥3 in 15 patients (35.7%). The details are presented in Figure 1.

**Figure 2:**
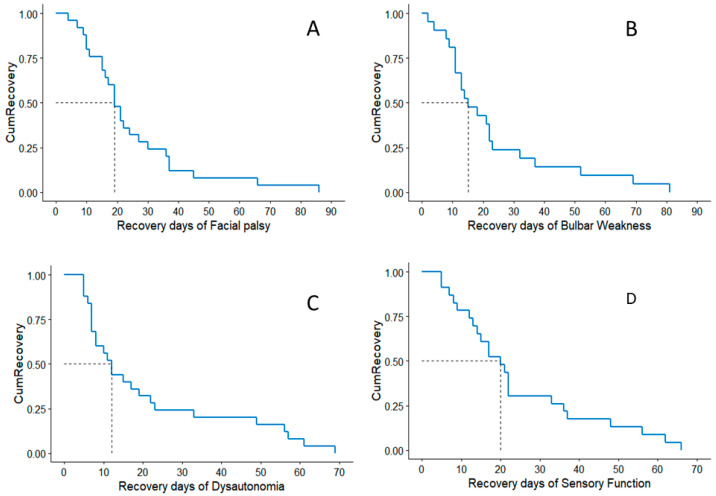
Days of complete recovery for (**A**) facial palsy, (**B**) bulbar weakness, (**C**) dysautonomia, and (**D**) sensory impairment.

Autonomic function recovered the earliest (median 12, range 5–69 days) followed by bulbar (median 15 days, range 2–81), facial (median 19 days, range 4–86 days), and sensory (median 20 days, range 5–66). Only two patients had a complete motor recovery by 20th day and 35th day, and in the remaining patients who had a good recovery (GBSDS ≤ 2), their median day of motor function recovery was 65 days (range 20–90). The time of complete recovery in different domains for neurological functions is presented in Figure 2.

The number of patients who recovered in different neurological functions in different subtypes of GBS is mentioned in Supplementary Table S2.

### 3.2. Comparison of Recovery Pattern between AIDP and AMAN

**Figure 3:**
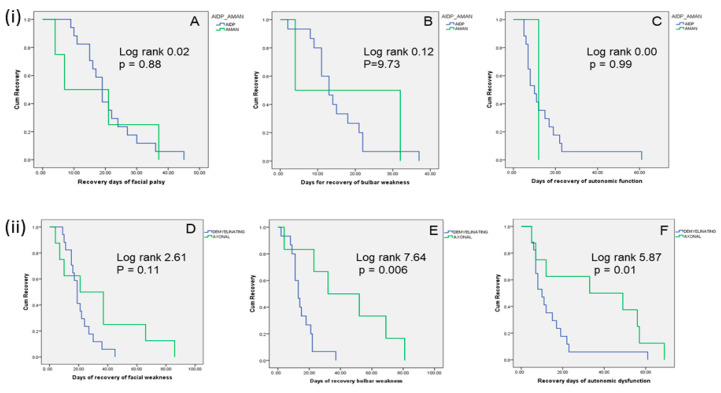
(**i**) Kaplan–Meier curve showing cumulative recovery of neurological functions at different time points between AIDP (acute inflammatory demyelinating polyradiculoneuropathy) and AMAN (acute motor axonal neuropathy). (**A**) Recovery in facial palsy, (**B**) recovery in bulbar palsy, (**C**) recovery in autonomic function. (**ii**) Kaplan–Meier curve showing cumulative recovery pattern of neurological functions between demyelinating and axonal subtypes of GBS. (**D**) Recovery in facial palsy, (**E**) recovery in bulbar palsy, (**F**) recovery in autonomic function.

The AIDP patients had more frequent dysautonomia (17.68% vs. 1.10%; *p* = 0.03), facial nerve palsy (19.76% vs. 4.40%; *p* = 0.06), and bulbar palsy (15.60% vs. 2.20%; *p* = 0.06) compared to AMAN. Other clinical and laboratory parameters were comparable (Supplementary Table S3). The days of complete recovery of bulbar, facial, autonomic, and motor deficits were comparable between AIDP and AMAN (Table 1). On Kaplan–Meier analysis, the day of recovery of facial (log rank 0.02; *p* = 0.88), bulbar (log rank 0.11; *p* = 0.73), and autonomic functions (log rank 0.00; *p* = 0.99) were not significantly different between AMAN and AIDP (Figure 3i).

### 3.3. Comparison of Recovery between Axonal and Demyelinating Subtypes

The demyelinating subtypes included patients with AIDP, equivocal and normal, and the axonal subtypes included AMAN, AMSAN, and IEMN. The patients with the axonal subtypes were hospitalised earlier than those with the demyelinating subtypes (5.41 ± 2.52 vs. 9.60 ± 7.97 days; *p* = 0.004). A higher proportion of patients with the demyelinating subtype had sensory impairment (22.88% vs. 5, 29.4%; *p* < 0.001). The recovery of bulbar function occurred earlier in the demyelinating group (*p* = 0.02) (Table 2).

The other clinical and laboratory parameters were not significantly different (Supplementary Table S4). However, on Kaplan–Meier analysis, the day of recovery of autonomic (log rank 5.87; *p* = 0.01) and bulbar function (log rank 7.64; *p* = 0.006) was significantly shorter in the demyelinating compared to axonal subtype (Figure 3ii).

### 3.4. Early Recovery

Four (9.5%) patients had an early recovery, and the proportion of patients with an early recovery in AIDP and AMAN, as well as the axonal and demyelinating types, was similar (). The early recovery was not dependent on IVIg or PLEX treatment (). (Supplementary Table S5) All the patients with AMSAN and IEMN had a late recovery.

## 4. Discussion

In this study on GBS, autonomic function recovered first, followed by bulbar, facial, and sensory function. Complete motor recovery took the longest time and occurred in 4.7% patients only. Axonal GBS took a longer time to recover than the demyelinating subtypes, irrespective of time to nadir and peak disability. This study for the first time reports the temporal profile of different domains of neurological recovery, and compares them between AMAN and AIDP, as well as the axonal and demyelinating subtypes. Several studies have reported predictors of short- and long-term outcomes, including age, autonomic dysfunction, respiratory failure, time to nadir, peak disability, axonal subtypes of GBS, and a low CMAP [12,13,20,21,22,23]. Almost all the studies, however, found time to nadir and peak disability to be independent predictors of GBS. Autonomic dysfunction and the requirement of MV are important predictors of a short-term outcome. The GBS disability scale is heavily dependent on motor function. Sensory impairment can only affect the GBSD scale if it is a pure sensory subtype with severe ataxia. Similarly, the MFS subtypes of GBS without weakness will also have walking difficulty if there is severe cerebellar ataxia.

Of the cranial nerve palsies, bulbar palsy recovered within a mean of 29.65 ± 29.10 days and facial palsy in 37.58 ± 37.65 days. The length of the facial nerve is 49–64 mm (the extratemporal part is 15–20 mm from the stylomastoid foramen to pes anserinus), and the motor fibres are thickly myelinated [24,25]. The length of the glossopharyngeal nerve is 32.6 mm ± 3.1 on the left side, 30.6 mm ± 3.7 on the right side, and the motor fibres are thickly myelinated [26]. The vagus nerve has a long course and is thinly myelinated. The exact site of facial nerve involvement in GBS is not known, but the most common site of facial palsy is at the stylomastoid foramen. In GBS, there is patchy focal demyelination or nodopathy extending from the spinal root to the most distal nerve. The recovery of cranial nerves not only depends on length but also on the extent of damage. The relative shorter length of the glossopharyngeal nerve may explain its earlier recovery than the facial nerve. The early recovery of autonomic function may be due to its thinly myelinated anatomical feature (<0.6 µ preganglionic sympathetic fibres) [27].

In different cohorts of GBS, a clinical sensory deficit has been reported in 35.3–64.1% patients [28,29]. Sensory loss is usually milder, involving the sense of fine touch and joint position impairment in distal lower limbs. A neurophysiological evaluation, however, detected sensory nerve conduction abnormality in 36–83.5% patients [30,31]. A sural-sparing pattern of sensory conduction has been reported, in which abnormal median and ulnar sensory conduction occurs with a normal sural sensory nerve conduction study [32]. In our earlier report on sensory conduction studies in 36 GBS patients, an abnormal median sensory conduction was noted in 21, ulnar in 17, and sural in 10 patients. An abnormal ulnar and sural NCS was always associated with a median NCS abnormality. A pattern of sural sparing was noted in 26 patients. Only one diabetic patient had an abnormal sural with a normal median and ulnar sensory NCS [33].

The thickness of myelin around the motor axon is greater (1.2–4.4 µ) than the sensory (0.2–0.8 µ) nerves [34]. The early recovery of sensory function compared to motor function in demyelinating GBS may be attributed to the anatomy of myelin. Axonal GBS has a different pathophysiology. Acute motor axonal damage may be triggered by *C. jejuni* infection. The primary immune-mediated injury in AMAN occurs at the axon without substantial T cell infiltration and demyelination. There is IgG and complement deposition in the nodal and internodal axolemma, and macrophage infiltration in the peri-axonal space. The recovery of axonal GBS depends on the extent of damage mediated by antiganglioside antibodies and the impairment of axonal sprout [35]. In AIDP, there is an infiltration of T cells and macrophages, leading to macrophage-mediated demyelination. In the present study, the recovery pattern of AIDP and AMAN in terms of facial, bulbar, autonomic, and motor function was similar. When compared the axonal and demyelinating subtypes, the recovery was slower in the axonal subtype. During the treatment course, many patients with AIDP and AMAN may shift to AMSAN and IEMN, respectively, due to secondary axonal changes [36]. Both IEMN and AMSAN took a longer time to recover, and independent walking was not achieved in 80% patients.

About 9.5% patients had an early recovery; the proportion of patients was comparable between AIDP and AMAN. Early recovery has been attributed to reversible conduction block and the clearance of antibody by treatment rather than remyelination or axonal regeneration. In a study on 80 patients with GBS, 11% patients had an early recovery. They often had AMAN (67%), retained tendon reflexes (44%), had anti-GM1 antibody (89%), and received IVIg (44%) [8]. In our study, the proportion of early recovery was not different between AMAN and AIDP or the axonal and demyelinating types, nor among IVIg, PLEX, or the natural course group.

The patients with complete motor recovery in our cohort were extremely low (4.7%), and may be due not only to the inclusion of normal muscle strength but also to normalisation of tendon reflexes. However, 35.7% of patients could walk unaided (GBSDS 1-2) at one month and 59% (GBSDS 1-2) of patients at three months. In a systematic review, the outcome of treated GBS patients at 4 weeks revealed death in 4%, the ability to walk unaided in 20.3%, and aided walking in 18% patients. Only 60% recovered full strength at one year [37]. Only 3 out of 10 studies in this review have mentioned 4 weeks outcomes. About 13.8–18.9% patients were able to walk unaided, and 11.9–66.7% required aid to walk [38,39,40]. In our earlier retrospective study including 388 patients, 42.8% patients had a complete recovery (GBSDS 0-1) at 3 months [41].

## 5. Limitations

This is a single-centre study, and it is based on a relatively small sample size. The clinical evaluation was performed by the same investigator (RM) under supervision of a senior neurologist (JK), thereby avoiding inter-rater variability. We have not assessed the antiganglioside antibodies, and the treatment was heterogeneous.

## 6. Conclusions

The neurological recovery in GBS occurs first in the autonomic, followed by the bulbar, facial, sensory, and motor functions. The recovery is comparable between AMAN and AIDP, although IEMN and AMSAN patients took a longer time to recover. The proportion of patients with an early recovery is similar in AMAN and AIDP.

## Figures and Tables

**Table 1:** Comparison of days of recovery of facial, bulbar, autonomic, and motor functions between acute demyelinating polyradiculoneuropathy (AIDP) and acute motor axonal neuropathy (AMAN) subtype within 3 months follow-up using nonparametric test.

Characteristics (Number of Patients Recovered)	AIDP N = 25	AMAN N = 10	*p* Value
Motor recovery days (2)	35.00	20.00	-
Cranial nerves			
Facial (25)	20.88 ± 9.41	17.25 ± 15.11	0.52
Bulbar (21)	15.13 ± 8.18	18.00 ± 19.79	1.00
Autonomic days (25)	14.29 ± 13.37	12.00 ± 0.00	0.78

**Table 2:** Comparison of days of recovery of facial, bulbar, autonomic, sensory, and motor functions between demyelinating and axonal subtypes of GBS within 3 months follow-up.

Characteristics (Number of Patients Recovered/Total)	Demyelinating N = 25	Axonal N = 17	*p* Value
Motor recovery days (2/42)	35.00	20.00	-
Sensory recovery days (23/28)	24.65 ± 18.17	Both AMSAN not recovered in 90 days	-
Cranial nerves			
Facial (25/29)	20.88 ± 9.41	33.50 ± 29.56	0.59
Bulbar (21/23)	15.13 ± 8.18	43.50 ± 29.12	**0.02**
Autonomic days (25/25)	14.29 ± 13.37	36.00 ± 25.32	0.10

## Data Availability

The data will be available on reasonable request from J.K. and R.M.

## Supplementary Materials

The following supporting information can be downloaded at: https://www.mdpi.com/article/10.3390/jcm13185635/s1, Table S1. Baseline characteristics. Table S2. Recovery of different neurological functions in different neurophysiological subtypes of Guillain-Barre syndrome. Table S3. Comparison of baseline characteristics of demyelinating and axonal types of Guillain-Barre syndrome (GBS). Table S4. Comparison of baseline characteristics of demyelinating and axonal types of Guillain-Barre syndrome (GBS). Table S5. Comparison of early recovery and late recovery groups.
