# Supplementary material for: Temporal Pattern of Individual Neurological Function Recovery in Guillain–Barré Syndrome
Source: J Clin Med. 2024 Sep 23;13(18):5635. doi: 10.3390/jcm13185635 (PMC11433427; doi:10.3390/jcm13185635)
Supplement: Supplementary file 1 [file jcm-13-05635-s001.zip › jcm-2895025-supplementary.pdf]

Journal of Clinical Medicine

Table S1. Baseline characteristics.

Characteristics	N=42
Age in years	35.05 ± 20.74
Female	11 (26.2 %)
Comorbidities	
Diabetes	1 (2.4%)
Hypertension	1 (2.4%)
Diabetes & Hypertension	5 (11.9%)
Others	8 (19%)
Duration of illness	7.9 ± 6.63
Admission days	
≤ 3 days	5 (11.9%)
4-7 days	25 (59.5%)
>7 days	12 (28.6%)
Antecedent illness	14 (56%)
Antecedent illness types	
URTI	7 (16.7%)
Diarrhoea	11 (26.2%)
Non-specific fever	5 (11.9%)
Malaria	1 (2.4%)
Peak disability (0-6 scale)	3.67 ± 0.79
Cranial nerves	
Facial	29 (69%)
Bulbar	23 (54.7%)
Others	6 (14.3%)
Dysautonomia	25 (59.5%)
GBS Clinical variant	
Sensorimotor	27 (64.3%)
Pure motor	14 (33.3%)
Miller-Fisher syndrome	1 (2.4%)
Electrodiagnostic subtypes	
AIDP	25 (59.5%)
AMAN	10 (23.8%)
AMSAN	1 (2.4%)
Equivocal	1 (2.4%)
IEMN	4 (9.5%)
Normal	1 (2.4%)
Mechanical Ventilator	7 (16.7%)
Treatment arm	
IVIg	28 (66.6%)
Plasmapheresis	3 (7.1%)
None	11 (26.1%)

AIDP = acute inflammatory demyelinating polyradiculoneuropathy, AMAN= acute motor axonal neuropathy, AMSAN= acute motor sensory axonal neuropathy, IEMN= inexcitable motor nerve, IVIg= intravenous immunoglobulin.

Table S2. Recovery of different neurological functions in different neurophysiological subtypes of Guillain-Barre syndrome.

Recovery @3months	AIDP (n=25)	AMAN (n=10)	AMSAN (n=1)	Equivocal (n=1)	Normal (n=1)	IEMN (n=4)
Motor	1/25	1/10	0/1	0/1	0/1	0/4
Sensory	23/25	-	0/1	0/1	0/0	0/1
Autonomic	17/17	1/1	1/1	1/1	1/1	4/4
Facial	17/19	4/4	0/1	1/1	0/0	3/4
Bulbar	15/16	2/2	1/1	0/0	0/0	3/4

AIDP = acute inflammatory demyelinating polyradiculoneuropathy, AMAN= acute motor axonal neuropathy, AMSAN= acute motor sensory axonal neuropathy, IEMN= inexcitable motor nerve.

Table S3. Comparison of baseline characteristics of demyelinating and axonal types of Guillain-Barre syndrome (GBS).

Characteristics	AIDP(N=25)	AMAN(N=10)	P value
Age	36.68 ± 21.97	25.90±14.41	0.16
Sex (F)	6(24%)	2(20%)	1.00
Comorbidities			
DM	1 (4%)	0 (0%)	
DM & HTN	3 (12%)	0(0%)	0.16
Others	7 (28%)	1 (10%)	
Duration of illness	9.60 ± 7.97	5.00 ± 2.16	0.08
Admission days			
≤ 3 days	1 (4%)	2(20%)	
4-7 days	15(60%)	7 (70%)	0.13
>7 days	9(36%)	1(10%)	
Antecedent illness	14 (56%)	5(50.0%)	1.00
Recurrent GBS	1 (4%)	3 (30%)	0.06
Peak disability (Hughes)	3.48 ± 0.65	3.40 ± 0.69	0.75
Cranial nerves			
Facial	19 (76%)	4 (40%)	0.06
Bulbar	15(60%)	2 (20%)	
Dysautonomia	17(68%)	1(10%)	**0.03**
GBS Clinical variant			
Sensorimotor	22 (88%)	1 (10%)	0.00
Pure motor	3(12%)	9 (90%)	
Mechanical Ventilator	2 (8%)	1 (10%)	1.00
Treatment arm			
IVIG	18 (72%)	4(40%)	
PLEX	3 (12%)	0(0%)	0.05
None	4 (16%)	6 (60%)	

AIDP = acute inflammatory demyelinating polyradiculoneuropathy, AMAN= acute motor axonal neuropathy, AMSAN= acute motor sensory axonal neuropathy, IEMN= inexcitable motor nerve, IVIg= intravenous immunoglobulin.

Table S4. Comparison of baseline characteristics of demyelinating and axonal types of Guillain-Barre syndrome (GBS).

Characteristics	Demyelinating N=25	Axonal N=17	P value
Age in years	36.68 ± 21.97	32.65 ± 19.8	0.54
Females	6 (24%)	5 (29.4%)	0.73
Comorbidities			
Diabetes	1 (4%)	0 (0%)	0.24
Hypertension	0 (0%)	1 (5.9%)	
Diabetes & Hypertension	3 (12%)	2 (11.8%)	
Others	7 (28%)	1 (5.9%)	
Duration of illness	9.6 ± 7.97	5.41 ± 2.52	**0.004**
Admission days			
≤ 3 days	1 (4%)	4 (23.5%)	0.13
4-7 days	15 (60%)	10 (58.8%)	
>7 days	9 (36%)	3 (17.6%)	
Antecedent illness	14 (56%)	10 (58.8%)	1.00
Peak disability (0-6 scale)	3.48 ± 0.65	3.94 ± 0.89	0.06
Cranial nerves			
Facial	19 (76%)	10 (58.8%)	0.31
Bulbar	16 (64%)	7 (41.2%)	0.34
Others	4 (16%)	2 (11.8%)	1.00
Dysautonomia	17 (68%)	8 (47.1%)	0.21
GBS Clinical variant			
Sensorimotor	22 (88%)	5 (29.4%)	**<0.001**
Pure motor	3 (12%)	11 (64.7%)	
Miller-Fisher syndrome	0	1 (5.9%)	
Mechanical Ventilation	2 (8%)	5 (29.4%)	0.09
Treatment arm			
Intravenous immunoglobulin	18 (72%)	10 (58.8%)	0.12
Plasmapheresis	3 (12%)	0	
None	4 (16%)	7 (41.2%)	

Table S5. Comparison of early recovery and late recovery groups.

	**Early Recovery (4)**	**Late recovery (38)**	**P**
AMAN (10)	2	8	0.56
AIDP (25)	2	23	
Demyelinating (25)	2	23	1.00
Axonal (17)	2	15	
IVIg	3	25	
PLEX	0	3	0.83
None	1	10	

IVIg= intravenous immunoglobulin, PLEX= plasmapheresis.
